# Use of the neutrophil-to-lymphocyte ratio as a component of a score to predict postoperative mortality after surgery for hip fracture in elderly subjects

**DOI:** 10.1186/s13104-016-2089-0

**Published:** 2016-05-26

**Authors:** Patrice Forget, Philippe Dillien, Harald Engel, Olivier Cornu, Marc De Kock, Jean Cyr Yombi

**Affiliations:** Department of Anesthesiology, Post-Anesthetic Outcome Unit, Université Catholique de Louvain, Av Hippocrate, 10-1821, 1200 Brussels, Belgium; Institute of Neuroscience (Pôle CEMO), Université Catholique de Louvain, Brussels, Belgium; Department of Orthopedic and Traumatologic Surgery, Université Catholique de Louvain, Brussels, Belgium; Department of Internal and Perioperative Medicine, Cliniques Universitaires Saint-Luc, Université Catholique de Louvain, Brussels, Belgium

**Keywords:** Outcomes, Orthopedics

## Abstract

**Background:**

Hip fracture precedes death in 12–37 % of elderly people. Identification of high risk patients may contribute to target those in whom optimal management, resource allocation and trials efficiency are needed. The aim of this study is to evaluate a predictive score of mortality after hip fracture in older persons on the basis of the objective prognostic factors easily available: age, sex and neutrophil-to-lymphocyte ratio (NLR) and C-reactive protein (CRP).

**Patients and methods:**

After the ethical committee approval, we analyzed our prospective database including 286 consecutive older patients (>64 years) with hip fracture. A score [range 0–4] was constructed, based on a previous analysis, combining age (1 point per decade above 74 years), sex (1 point for male gender) and NLR at postoperative day +5 (1 point if > 5). A receiver-operating curve (ROC) analysis was performed. Similar analyses were performed with CRP (1 point if > 7.65 mg/dL).

**Results:**

In the 286 patients (male 31 %), the median age was 84 (65–102) years, and the mean NLR values were 6.47 ± 6.07. At 1 year, 82/286 patients died (28.7 %). In the 235 patients with complete data, significant differences in term of mortality risk are observed (*P* < 0.001). Performance analysis shows an AUC of 0.72[95 % CI 0.65–0.79]. CRP performed less than NLR (AUC for CRP alone: 0.53[95 % CI 0.45–0.61], *P* = 0.42, with a sensitivity of 58.5 % and a specificity of 57.1 % for a cut-off value of 7.65 mg/dL; and for NLR alone: 0.59 [95 % CI 0.51–0.66]; *P* = 0.02, with a sensitivity of 55 % and a specificity of 65 % for a cut-off value of 4.9).

**Conclusion:**

A discrete 0–4 scoring systems based on age, sex and the NLR was shown to be predictive of mortality in elderly patients during the first postoperative year following surgery for hip fracture repair.

## Background

Hip fracture precedes death in 12–37 % of elderly people [1]. While its high incidence, identification of high risk patients in whom optimal management and resource allocation remains a problem [2].

To use a biomarker as a predictor could be an interesting way to address this question. The neutrophil-to-lymphocyte ratio (NLR) has been proposed in other postoperative context [3–5]. A previous study of our team shows that the NLR at the fifth postoperative day (D5) is an important prognostic factor considering mortality risk after hip fracture [6]. Nevertheless, its usefulness is limited as a predictor of mortality when considered alone. Consequently, it may be a good potential candidate if considered as included in a composite score. Age and sex, shown in several reports as important objective prognostic factors [7, 8] are also good candidates to include in a score that could be widely used and easily compared between centers.

Based on a previous work [6], the aim of this study is to evaluate a predictive score of mortality after hip fracture on the basis of these objective variables (age and sex), to evaluate its performance in the elderly patients of our hip fracture registry, and to consider the added value of the NLR (at D5, defined as 5 days after surgery) to the score. A second aim is to consider the potential performance of another inflammatory biomarker, the C-reactive protein (CRP), as an alternative to the NLR.

## Patients and methods

### Patients

After the approval of the ethical committee (CEBHF of the Université catholique de Louvain, Chairperson: Pr J.-M. Maloteaux, n°2010/23DEC/406), that waived us from written informed consent for this observational study, we analyzed our prospective database including 286 consecutive patients, undergoing surgery for hip fracture, from September 2010 to February 2012. These data were registered and managed by the physician in charge of the patients (JCY) in agreement with the Belgian law.

### Design and data collection

Data collection was systematized and standardized using computerized medical charts. Age, sex, comorbidities, NLR and CRP values were registered. Regarding the NLR value, we introduced it as a binary variable (NLR > 5 or not) as proposed by Proctor et al. [9]. Indeed, we assumed the possibility of a non-linear impact of the NLR-value on outcome. To manage the risk of an empirical cut-off, proposed by Proctor et al, we completed previously the analysis by a pre-planned ROC curve analysis. This analysis permitted to confirm that the most discriminant NLR value was 4.9, associated with a sensitivity of 62.9 % and a specificity of 57.6 % for mortality prediction (P = 0.01) [6]. This findings permitted to use the Proctor et al’s threshold (NLR > 5) and to consider it for subsequent analyses.

Survival data were obtained from the Belgian national registry, in accordance to the national laws, permitting a complete, and high-quality, follow-up. Cause of death is not investigated. In this series, we found similar risk factors for mortality than in the literature: age, male gender and multiple comorbidities (defined here as: cirrhosis, arterial hypertension, COPD, vascular disease, coronaropathy, other cardiomyopathy—including chronic heart failure, diabetes mellitus, dementia, anaemia necessitating blood transfusion) [6]. Compared to the literature, the various definitions of the multiple comorbid status precludes definitive comparison. Therefore, we limited our model to objective risk factors.

### Objectives

Our first objective is to construct and evaluate the performance of a predictive risk score, based on the three most important and objective risk factors for mortality identified in our cohort of patients >64 years with hip fracture: age, sex and NLR > 5 at D5 after surgery [6].

Our second objective is to compare, in this context, the potential performance of the CRP, as a potential alternative to the NLR, as prognostic significance of CRP was not analyzed in our previous report.

### Planification of surgery and postoperative care

All the patients were taken in charge following the same protocol, including early surgery [10] (87.7 % of surgeries were done within the first 24 h, 95.4 % within the first 48 h). All surgeries were performed by the same team, coordinated by OC. As recommended, clinical follow-up was made by a multidisciplinary medical team (surgeons, geriatricians, anesthesiologists, with a coordinating general internist, JCY) [11].

### Blood analyses

Blood analyses were performed as following. All venous blood samples were processed in a blood analyzer [Sysmex (TOA Medical Electronics, Kobe, Japan)] for the determination of the complete blood cell counts and differential counts of leukocytes. We recorded the neutrophils and the lymphocytes counts, and calculated the NLR. The CRP was determined by turbidimetry [UniCel^®^ DxC 800 (Beckman Coulter, Pasadena, California, USA)] on a serum sample.

### Statistical analysis

Data are presented as mean ± SD, number (percentage) and median (range) or percentage [95 % confidence interval, CI]. Factors considered here for the construction of the score were selected as the three most important objectives ones previously identified [6]. The coefficients associated with these factors were used to construct a predictive score.

### Construction of a predictive score

A methodology, derived from this used previously by Apfel et al. [12], was used. The following formula permitted risk probability modelisation = (1 + e^−z^)^−1^ with z = b_1_ . x_1_ + b_2_ . x_2_ + b_3_ . x_3_ + … + b_y_ . x_y_ with b as the coefficient calculated in the regression model and x the parameter. For this work, the following coefficients were obtained from a previous regression model (the hazard ratio–HR—for the mortality risk) [6]: Age by 10 year-increments: 2.08 [95 % CI 1.37–3.17]; male gender: 1.92 [95 % CI 1.17–3.14] and NLR > 5 at day 5: 1.8 [95 % CI 1.11–2.94] (*P* < 0.05 for all the analyses concerning mortality risk at 1 year). Taking into account that no additional risk of death correspond to 1, b was obtained considering the HR − 1 with b_1_ = 1; b_2_ = 1 and b_3_ = 1.

A receiver-operating curve (ROC) curve analysis was planned to determine the performance of the score, by reporting the area under the curve (AUC). Result was considered by some authors as associated with correct performance if equal or more than 0.7 [13]. Another ROC curve analysis was planned to compare the performance of the CRP and this of the NLR values, as without these markers. For survival analysis, Kaplan–Meier analyses were used with log-rank test. In all the analyses, *P* ≤ 0.05 was considered as significant. Analyses were performed using the software STATISTICA (7, Statsoft Inc. 2004) and SPSS Statistics (17.0, Polar Engineering and Consulting 2008).

## Results

### Patients

From the 286 patients included, 235 were retained in the final analyses. Causes are lack of NLR and/or CRP data at D5 (n = 49, 17.1 %) and lost-of-follow-up due to departure to another country (n = 2, 0.7 %). In these 235 patients (72 males and 163 females, 30.6 %/69.4 %), median age is 84 (range: 65 to 102) years. Mean NLR values at day 5 are 6.47 ± 6.07. Proportion of patients with a NLR at D5 > 5 is 46.0 % (n = 108/235). At 1 year, 82/286 patients died (28.7 %) [14]. These proportions were similar in the 51 patients excluded (19 males and 32 females, 37.2 %/62.7 %), with a median age of 84 (range: 65–96) years, and mortality at 1 year of 15/49 (30.6 %) (*P* > 0.05 for all comparisons with patients retained with the final analyses).

### Construction of a predictive score

Based on the previously observed coefficient factors, age is considered as decades in the score (0 point from 65 to 74 years, 1 point from 75 to 84 years and 2 points above 84 years). One point is added for males. To see whether these risk factors are sufficient to construct a score (ranging from 0 to 3), we did a performance analysis of this score that reveals no predictive value, with an AUC of 0.52 [95 % CI 0.43–0.60] (*P* = 0.69 *vs.* AUC = 0.5).

**Fig. 1:**
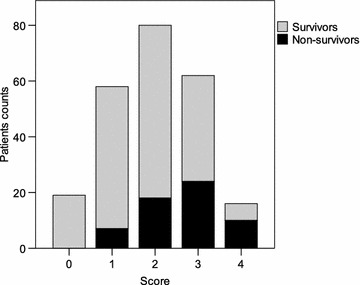
Score distributions the 235 patients of more than 64 years after surgery for hip fracture. Score is based on sex (1 = male), age (1 = more than 74 years, 2 = more than 84 years) and NLR at day 5 (1 if NLR > 5). Survivor/non-survivor status were assessed at one year

**Fig. 2:**
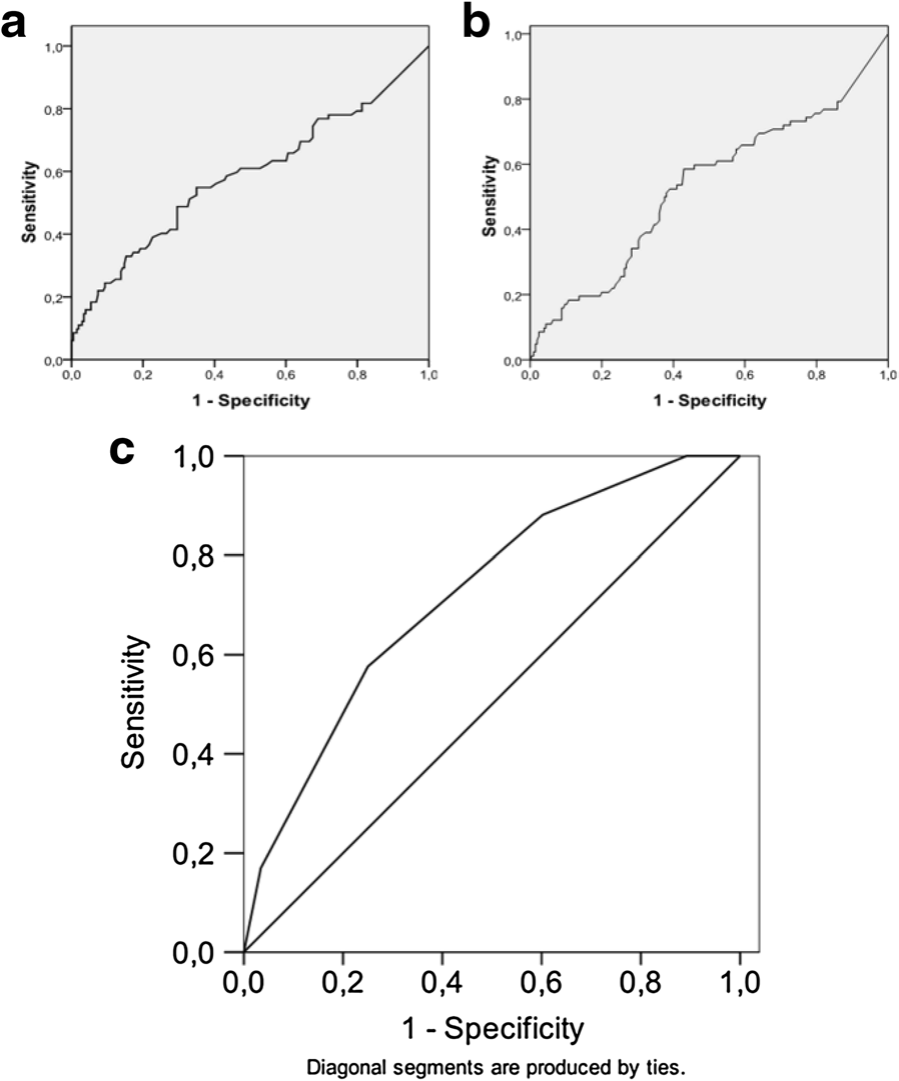
Performance analysis of a the NLR (**a**), the CRP (**b**) values, or a predictive score (**c**) for mortality at one year in a series of 235 patients of more than 64 years after surgery for hip fracture. Score is based on sex (1 = male), age (1 = more than 74 years, 2 = more than 84 years) and NLR at D5 (1 if > 5). Areas under the curve (AUC) are, for NLR, 0.59 [95 % CI 0.51–0.66](*P* = 0.02 *vs.* AUC = 0.5) with an with an optimal cut-off value of 4.9 (**a**); for CRP, 0.53 [95 % CI 0.45–0.61](*P* = 0.42 *vs.* AUC = 0.5), with an optimal cut-off value of 7.65 mg/dL (**b**), and, for the composite score, 0.72 [95 % CI 0.65–0.79] (*P* < 0.001 *vs.* AUC = 0.5)

As planned, NLR > 5 at D5 after surgery was considered in the score, with one additional point when positive. Then, a score ranging from 0 to 4 is obtained for all the patients (Table 1). Distributions of the score in all the series [median: 2 (0 to 4)], in survivors [2 (0 to 4)] and non-survivors [3 (1 to 4)] are presented in Fig. 1. Performance of the test analysis shows an AUC of 0.72 [95 % CI 0.65–0.79] (*P* < 0.001) (Fig. 2). This score is therefore considered in subsequent analyses.

**Table 1:** Proposed score, ranging from 0 to 4, to predict mortality after hip fracture

Age (years)	Sex	NLR > 5 at postoperative day 5
65 to 74 = 0	Male = + 1	If yes = + 1
75 to 84 = + 1	Female = 0	
Above 84 = + 2		

Based on the previously observed coefficient factors, age is considered as decades in the score (0 point from 65 to 74 years, 1 point from 75 to 84 years and 2 points above 84 years). One point is added for males. Performance analysis of this score, ranging from 0 to 3, reveals no predictive value, with an AUC of 0.52 [95 % CI 0.43–0.60] (*P* = 0.69 *vs.* AUC = 0.5)

As planned, NLR > 5 at D5 after surgery was considered in the score, with one additional point when positive. Then, a score ranging from 0 to 4 is obtained for all the patients

### Mortality at 1 year in regards to the score value

**Fig. 3:**
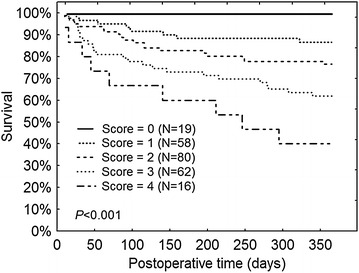
Overall survival curves at one year (Kaplan–Meier analysis) in a series of 235 patients of more than 65 years after surgery for hip fracture. Score is based on sex (1 = male), age (1 = more than 74 years, 2 = more than 84 years) and NLR at D5 (1 if > 5)

Cumulative survival curves are presented in Fig. 3. Graphical analysis confirms a gradual increase in the mortality risk associated with the predictive score. Log-rank test confirms a highly statistically significant difference (*P* > 0.001).

### CRP *vs.* NLR

Mean CRP values at day 5 are 10.32 ± 7.02. The AUC are 0.53 [95 % CI 0.45–0.61] (*P* = 0.42 *vs.* AUC = 0.5) and 0.59 [95 % CI 0.51–0.66](*P* = 0.02 *vs.* AUC = 0.5) respectively for CRP and NLR. Optimal cut-off values are 7.65 mg/dL, for the CRP, and 4.9 for the NLR. Proportion of patients with a CRP at D5 > 7.65 mg/dL is 54.9 % (n = 129/235). Consequently, unsatisfactory (i.e. <0.7) AUC values, concerning 1-year mortality, with the CRP and the NLR taken alone are confirmed. In addition, results show that CRP shows no potential interest in a composite score in contrast with the NLR (*P* < 0.05 *vs.* AUC = 0.5).

## Discussion

We constructed and tested here a predictive score for mortality at 1 year after hip fracture in elderly patients (>64 years). The score comes from three objective parameters available at D5 after surgery: sex, age and NLR > 5, giving a value from 0 (a woman, between 65 and 74 years, with a NLR < 5 at D5) to 4 (a man, older than 84 years, with a NLR > 5 at D5). This score presents a predictive performance, with an AUC of 0.72 [95 % CI 0.65–0.79]. In our analyses, CRP does not show any advantage on NLR. It is true when comparing directly CRP to NLR performances, and when included in the score.

There are already models in the literature like the Charlson comorbidity index (CCI). But, in their study, Neuhaus et al. [15] conclude that, while the CCI predicted in-hospital mortality in patients with hip fractures, other factors may be of value in patients with trauma. The CCI has been developed for patients without trauma [16] and it is based on comorbidities [17], parameters which we do not consider in our score, as our approach aimed to restrict us to objective parameters. Furthermore we want a score to predict mortality when the patients have left the hospital and our score can be seen as simpler as based on only three factors. However, our score lacks of major advantages of the CCI: excellent performance (C statistics of more than 0.85) and multiple validations in many different centres.

The Nottingham hip fracture score developed by Maxwell et al. [18] is also interesting. This score originally intended to measure mortality at 30 day (and mostly validated for). In a second study, it was showed that it could also be used to predict 1-year mortality after HF [19]. However it takes into account many parameters and cannot be easily calculated at the bedside without the help of electronic tools. For the same reason we were not satisfied with the score created by Jiang et al [20].

Concerning the importance of the NLR in the score, it can be said that the acute, as the persisting (possibly in patient with preexisting vascular disease), inflammatory response observed after a vascular lesion or an ischemic event is, at least partially, activated by activated neutrophils [21–23]. With platelets, neutrophils participate to endothelial dysfunction, the destabilization of atherosclerotic plaques and coagulation enhancement, inducing further vascular damage [24, 25]. These effects are dependent of the magnitude and the duration of the response [26, 27]. This does not exclude the possibility is that NLR on day 5 is a marker of frailty, with a stress-induced hormonal changes includes cortisol secretion, which increases the neutrophil count and reduces the lymphocyte count. All these possibilities may contribute to the fact that mortality at 1 year is higher in patients with a NLR > 5 at D5.

Limits are linked to the design of our work that can be considered as an internal validation of the study score. Indeed, the data serving to identify the prognostic factors were, at least partially, dependent from these serving to test the score (i.e. coming from a previous version of the same registry). Nevertheless, as the chosen parameters are objective (sex, age, NLR at D5), an external validation in another institution will be easy to perform. One important bias could be the exclusion of the 49 patients with incomplete data. However, all the relevant data (risk factors) were similar between included and excluded patients, especially survival (*P* > 0.05). Finally, to increase the usefulness of the score, it will be interesting to compare it with other methods and scores. Indeed, as the score is obtained only at D5, it is not sure whether normal clinical experience would be not as well estimated. Moreover, an AUC of 0.72 underlies a significant proportion of false positive and negative results.

## Conclusion

We have developed a score to predict the risk of mortality at 1 year in elderly patients after surgery for a hip fracture. The score is based on age, sex and the NLR at D5. After external validation, it may be included in clinical practice as in clinical research to stratify the risk of postoperative mortality.
